# FOXL2 down-regulates vitellogenin expression at mature stage in *Eriocheir sinensis*

**DOI:** 10.1042/BSR20150151

**Published:** 2015-12-22

**Authors:** Qing Li, Jing Xie, Lin He, Yuanli Wang, Hongdan Yang, Zelin Duan, Qun Wang

**Affiliations:** * Laboratory of Immunological Defense & Reproduction, School of Life Science, East China Normal University, Shanghai 200241, China

**Keywords:** DDX20, FOXL2, FTZ-F1, follicular cells, mature stage, vitellogenin (VTG) synthesis

## Abstract

Ovarian development in crustaceans is characterized by rapid production of egg yolk protein in a process called vitellogenesis. In the present study, we investigated the involvement of a DEAD (Asp-Glu-Ala-Asp) box RNA helicase 20 (DDX20), forkhead transcription factor (FOXL)2 and fushi tarazu factor (FTZ-F)1 in the regulation of vitellogenesis. Based on ESTs from the testis and accessory gland of *Eriocheir sinensis*, we cloned the full-length cDNAs of *foxl2* and fushitarazu factor 1 (*ftz-f1*), which include the conserved structural features of the forkhead family and nuclear receptor 5A (NR5A) family respectively. The expression of *foxl2* mRNA surged at the mature stage of the ovary, when *vtg* mRNA swooped, suggesting that *foxl2* negatively affects the vitellogenin (VTG) synthesis at this developmental stage. Etoposide (inducing germ cell apoptosis) treatment up-regulated FOXL2 and DDX20 at both the mRNA and the protein levels, primarily in the follicular cells as shown by immunofluorescence analysis. Furthermore, *foxl2*, *ddx20* and *ftz-f1* mRNA levels increased significantly with right-eyestalk ablation. Interactions between FOXL2 and DDX20 or FTZ-F1 were confirmed by co-immunoprecipitation and the forkhead domain of FOXL2 was identified as the specific structure interacting with FTZ-F1. In conclusion, FOXL2 down-regulates VTG expression by binding with DDX20 in regulation of follicular cell apoptosis and with FTZ-F1 to repress the synthesis of VTG at the mature stage. This report is the first to describe the molecular mechanism of VTG synthesis in *E. sinensis* and may shed new light on the regulation of cytochrome P450 enzyme by FOXL2 and FTZ-F1 in vitellogenesis.

## Abstract (precis)

The present study highlights that forkhead transcription factor (FOXL)2 down-regulates vitellogenin (VTG) synthesis not only through the regulation of follicular cell apoptosis with DEAD-box RNA helicase 20 (DDX20), but also may through the steroidogenic pathway with fushi tarazu factor (FTZ-F)1 at mature stage in *Eriocheir sinensis*.

## INTRODUCTION

As with other oviparous animals, ovarian development in crustaceans is characterized by rapid production of egg yolk protein in a process called vitellogenesis [1]. Vitellogenesis refers to the synthesis of vitellogenin (VTG), the precursor for the major yolk protein vitellin (VT) and further accumulation of yolk body and lipid droplets in oocytes [2]. In non-mammalian vertebrates [3] and several invertebrates [4], VTG is produced in an extra-ovarian tissue and then transported into the ovary. In *Eriocheir sinensis*, yolk protein originates from two sources: endogenous yolk that comes from VTG synthesized in the endoplasmic reticulum and Golgi apparatus of oocyte at early vitellogenesis and the exogenous yolk which the oocyte incorporates VTG directly from the haemolymph through the surrounding follicular cells at late vitellogenesis [5,6]. With VTG continuously transported into the oocytes, the follicular cells acted as a ‘bridge’ thinning gradually and becoming connective tissue with no cell structure [6]. Notably, follicular cells are thought to be crucial in the transportation of VTG at late vitellogenesis.

Defects in folliculogenesis are reported to account for 50.7% of the total incidence of premature ovarian failure (POF) [7]. In the ovary, forkhead transcription factor gene 2 (*foxl2*) is involved in the regulation of cholesterol and steroid metabolism, apoptosis, detoxification of reactive oxygen species and cell proliferation [8]. In mice, FOXL2 is mainly expressed in undifferentiated granulosa cells and follicular cells surrounding oocytes and mutations of FOXL2 can damage these cells, terminating the developmental process of follicular cells. As a result, the insufficient synthesis of cell division inhibitory factors in theca and granulosa cells can lead to the degradation of oocytes after the first meiotic division, eventually causing POF [9]. Follicular cells are pivotal in vitellogenesis of follicle-type species. In *Penaeus merguiensis* [10] and *Charybdis japonica* [11], follicular cells transport VTG into oocytes. In addition, follicular cells have the ability to synthesize VTG in *Macrobranchium rosenbergii* [12]. Previously, FOXL2 was demonstrated to induce apoptosis in both Chinese hamster ovary cells and rat granulosa cells by interacting with DDX20, a DEAD-box (Asp-Glu-Ala-Asp box) protein [13]. As germ cells are highly sensitive to DNA damage [14,15], in this study, we treated crabs with etoposide, a podophyllotoxin semi-derivative agent used in chemotherapy, to induce ovarian apoptosis and explore the function of FOXL2 and DDX20 in ovary development.

Furthermore, vitellogenesis in crustaceans is also regulated by various related endocrine hormones as in vertebrate species. Ecdysteroids, which is one kind of steroid hormones that regulate growth, development, reproduction and molting in arthropods, can stimulate fat bodies to produce VTG [16]. FOXL2 also functions as a transcriptional repressor of several key genes involved in biosynthesis of steroid hormones, including the human steroidogenic acute regulatory protein (STAR) gene, which translocate cholesterol from the outer to the inner membrane of mitochondria, the rate-limiting step in steroidogenesis. In rats, FOXL2 can interact with SF-1 [steroidogenic factor 1, the vertebrate homologue of the insect fushi tarazu factor (FTZ-F)1] and repress SF-1-induced *CYP17* transcription in granulosa cells [17]. In *Nile tilapis*, FOXL2 has been demonstrated to interact through its forkhead domain with the ligand-binding domain of SF-1 to form a complex and enhance the SF-1-mediated *CYP19a1* transcription [18]. These findings raise the possibility that FOXL2 may interact with fushitarazu factor 1 (FTZ-F1) to regulate the transcription of cytochrome P450 enzyme and indirectly involved in VTG synthesis. However, similar molecular studies on VTG synthesis in crustaceans are lacking.

The Chinese mitten crab is an important aquaculture species in Southeast Asia, with important food and economic value [19] and is also often employed as model organism of *Brachyura* in reproductive studies [19–24]. Accumulation of yolk protein is related to their reproduction and larval quality. Unfortunately, no studies could furnish any direct evidence to prove the role of FOXL2 in the regulation of VTG synthesis. In present study, we aimed to explore the molecular mechanism of FOXL2 in the regulation of VTG synthesis.

## MATERIALS AND METHODS

### Experimental animals

Healthy adult Chinese mitten crabs were purchased from a Shanghai aquaculture farm from July 2012 to January 2014. Eighteen crabs of September were injected intramuscularly with different concentrations of etoposide (0, 30, 60, 90, 120, 150 μM) and killed after 24 h, 15 were injected intramuscularly with 60 μM etoposide and killed at different time points (0, 6, 12, 18 and 24 h). In addition, crabs purchased in September of 2014 were divided into four groups, with four healthy crabs in every group. The right eyestalk, each of 16 crabs, was ablated (ESA: eyestalk ablation) at the same time according to a previously described method [25] and killed at 0, 1, 3 and 6 day later respectively. Animal experiments conducted were approved by the Ethics Committee of Laboratory Animal Experimentation at East China Normal University.

### Samples preparation

After haemolymph was taken according to the previously described method [25], tissues (i.e. vas deferens, hepatopancreas, heart, muscle, stomach, ovary, testis, accessory gland, thoracic ganglia, intestine and seminal vesicle; ovary, testis and accessory gland with etoposide; ovary with ESA) were resected from individuals, frozen immediately in liquid nitrogen and stored at–80°C until used for RNA extraction, western blot and immunoprecipitation analysis.

According to the ovarian tissue histological study of *E. sinensis* by Xue et al. [26], the ovaries are divided into the following four stages based on the development state of the first batch of oocytes and colour of ovaries: oogonium stage (April–July), ovaries are transparent milky white; primary oocyte niche for a long time stage (August–September); primary oocyte growth period (October–November), ovaries are light brown coloured and the vitellogenesis process occurs in this stage; mature stage (December–March), ovaries are dark red-brown coloured. Crabs from July, October and December represented mainly three stages of ovarian development were anaesthetized for 3–5 min and dissected immediately to obtain the ovary. Fresh ovarian tissues were fixed in 4% paraformaldehyde for immunofluorescence analysis.

### Primary culture and treatment of *E. sinensis* ovarian tissue

Ovarian tissues from individuals of September were isolated and washed several times in PBS (phosphate buffer solution) (pH 7.4, CWBIO) containing 100 units/ml penicillin (Gibco) after they were soaked in 1% potassium permanganate for 0.5 h and surface disinfected with 75% alcohol. The tissues were then cut into 1 mm^3^ sections and placed in 24-well culture plates (Gibco), with each well containing 2 ml of M199 medium (20% FBS (fatal bovine serum), 100 units/ml penicillin, 100 μg/ml streptomycin and 50 μg/ml kanamycin; Gibco) and etoposide at various concentrations (0, 30, 60, 90, 120 and 150 μM, Novartis Pharma). Tissues were then incubated for 24 h at 26°C–27°C [27–29]. Each experiment designed three parallel controls. All ovarian tissues were fixed and cut into 20-μm thick sections and analysed by immunofluorescence.

### RNA extraction

Total RNA was isolated from frozen tissues using TRIzol (Invitrogen) according to the manufacturer's instructions, quantified based on the absorbance at 260 nm and checked for integrity by agarose gel electrophoresis.

### Cloning the full length of *foxl2*, *ftz-f1* and partial *ddx20* cDNAs in *E. sinensis*

The partial cDNA fragments of *foxl2*, *ftz-f1* and *ddx20* were obtained from the transcriptome analysis of testes and accessory gland in Chinese mitten crab [30,31]. We first confirmed the sequences by PCR using specific primers (Table 1) and then rapid amplification of cDNA ends (RACE) was performed to obtain the full length of the *foxl2* and *ftz-f1* genes under the manufacturer's instruction (SMART™ RACE cDNA Amplification kit, Clontech). The primer sequences used are listed in Table 1. The PCR programme of 5′ race was run as follows: 94°C for 4 min, 94°C 30 s, *T*_m_ (annealing temperature)=65°C for *foxl2*, 60°C for *ftz-f1* 40 s, 72°C 30 s, for seven cycles with a 1.0°C decrease at annealing temperature per cycle; 94°C 30 s, 59°C/54°C 30 s,72°C 30 s, for 20 cycles; then 10 min at 72°C for the ending extension; incubated at 4°C. The PCR programme of 3′ race was carried out as follows: 94°C for 4 min, 94°C 30 s, *T*_m_=68°C/61°C 30 s, 72°C 45 s, for six cycles with a 1.0°C decrease at annealing temperature per cycle; 94°C 30 s, 62°C/55°C 30 s, 72°C 45 s, for 25 cycles; then 10 min at 72°C for the ending extension; incubated at 4°C.The purified PCR products were cloned into the pZeroBack/blunt vector and transformed into *Escherichia coli* Top10 competent cells. The potentially positive recombinant clones were identified by colony PCR and picked for sequencing.

**Table 1:** Primers used in the experiments

	Primer name	Sequence
Specific primers for target *genes*	*foxl2* Forward primer	5′-GGGGACCTGGACCCTAACAA-3′
	*foxl2* Reverse primer	5′-GGAAACATTCGTTCAGACTCAGGT-3′
	*foxl2* 5′ race primer	5′-TGTTAGGGTCCAGGTCCCCGTTCTC-3′
	*foxl2* 3′ race primer	5′-CGGCACAACCTGAGTCTGAACGAATG -3′
	*ddx20* Forward primer	5′-CCGAATAAGGCTGCCGCTGTGAT-3′
	*ddx20* Reverse primer	5′-AAGAGTGGACCAGACCTGAGCATAGAG-3′
	*ftz-f1* Forward primer	5′-AGGTTGATGACCAGATGAAGC-3′
	*ftz-f1* Reverse primer	5′-TTCCGTTTAGTGTGGAGCAT-3′
	*ftz-f1* 5′ race primer	5′-GGCAATGAAGTGGAGTTCGGGGAGC-3′
	*ftz-f1* 3′ race primer	5′-CTGCTCCCCGAACTCCACTTCATTGC-3′
qRT-PCR primers	*foxl2* Forward primer	5′-GGGGACCTGGACCCTAACAA-3′
	*foxl2* Reverse primer	5′-GGAAACATTCGTTCAGACTCAGGT-3′
	*ddx20* Forward primer	5′-GAAGAAGCAGAAGACTGGAGGGA-3′
	*ddx20* Reverse primer	5′-GAGGAAGAACACTGGGTGGAAG-3′
	*ftz-f1* Forward primer	5′-TTGCTGATTCTTGACCACCT-3′
	*ftz-f1* Reverse primer	5′-GGTATTTTTCACCGTTGTCG-3′
	*vtg* Forward primer	5′-CAGCGAACAAAAGAAAGTG-3′
	*vtg* Reverse primer	5′-CTGTCAGATAGGCAGCCAAT-3′
*β-actin* and *GAPDH* primers for qRT-PCR	β-actin-F	5′-CTCCTGCTTGCTGATCCACATC-3′
	β-actin-R	5′-GCATCCACGAGACCACTTACA-3′
	GAPDH-F	5′-TGGTGGAGCCAAGAAGGTG-3′
	GAPDH-R	5′-ACGGGAGCCAGGCAGTT-3′
Primers for forkhead domain of *Es-FOXL2*	*foxl2* Forward primer	5′-CCGGAATTCATGGCGATCAAGGAGAGC-3′
	*foxl2* Reverse primer	5′-CCGCTCGAGTTAATAGGGAGGGTAGGGCTG-3′
Sequencing	T7 primer	5′-TAATACGACTCACTATAGG-3′
	SP6 primer	5′-ATTTAGGTGACACTATAGAA-3′

### Bioinformatic analysis

Sequences analyses were performed using the Blastprogram at the National Center for Biotechnology Information (http://www.ncbi.nlm.nih.gov/blast). ORF finder (www.ncbi.nlm.nih.gov/gorf/gorf.html) was used to determine the ORF. The ExPASy ProtParam tool (http:://web.expasy.org/protparam) was used to analyse the molecular mass and isoelectric point. Multiple sequence alignment of protein was performed using the ClustalX Multiple Alignment program and DNAman software. The phylogenic tree was built using the neighbour-joining (NJ) method of MEGA version 4.0 software with 1000 bootstrap replications.

### Quantitative real-time reverse transcription PCR

Total RNA (2 μg) for each sample was subjected to DNase treatment (70 units/μl, Takara) to eliminate gDNA (genome DNA) contamination. Using the One Step SYBR PrimeScript reverse transcription PCR (RT-PCR) kit (Takara), 0.5 μg/μl of each RNA sample was reverse transcribed to cDNA with the resulting cDNA diluted 1:10 RNase-free H_2_O for use in subsequent real-time PCR. Specific primer pairs for *foxl2*, *ftz-f1*, *ddx20* and *vtg* (Genbank accession number: AY910692; Table 1) were used to amplify products of 210, 135, 150 and 149 bp respectively, which were sequenced to verify the specificity. *β-actin* (HM053699.1) and *GAPDH* (glyceraldehyde-3-phosphate dehydrogenase; HM053701.1) from *E. sinensis* were used to calibrate the cDNA template as internal controls. Quantitative real-time RT-PCR (qRT-PCR) was carried out on a CFX96 real-time PCR detection system (Bio-Rad) and each sample was run in triplicate in a total volume of 25 μl with 12.5 μl of SYBR Premix Ex Taq II (2×), 1 μl of each primer (10 μM),1 μl of cDNA template and 9.5 μl of double distilled water (ddH_2_O). The PCR programme was 95°C for 3 min, followed by 40 cycles of 95°C for 10 s, 60°C for 30 s, 65°C for 5 s and 95°C for 5 s. Dissociation curve analysis was performed at the end of each PCR reaction to verify the amplification of a single product. The 2^−ΔΔCt^ method was used to determine fold change for the gene expression relative to controls [32]. All the experiments were independently repeated three times and the results were the average of triplicate determinations.

### Plasmid construction and expression of recombinant pET-32a-EsFH plasmid in *E. coli*

The cDNA fragment encoding the forkhead domain of FOXL2 was amplified with specific primers (Table 1) to produce a 198-bp product, which was inserted into the pET-32a vector between EcoRI and XhoI restriction sites to generate the forkhead domain expression construct. The constructed plasmid was designated as pET-32a-EsFH. The *E. coli* BL21 containing the recombinant pET-32a-EsFH plasmid was inoculated into LB liquid medium with 100 μg/ml ampicillin (Amp). When the *A*_600_ reached 0.8–1.0, IPTG was added at different final concentrations (0.4, 0.6, 0.8, 1.0, 1.2 mM) to induce the fusion protein for different periods (2, 3, 4, 5 and 6 h) at 37°C. The expression level of recombinant protein was evaluated by SDS/PAGE and stained by Coomassie Blue R-250.

### Western blot

The total protein (500 μg) was extracted and loaded on a SDS/PAGE (12% gel). After separation, the proteins were transferred to a methanol-activated polyvinylidene fluoride membrane (CWBIO) by electroblotting. The membrane was blocked with 5% BSA in 10 mM PBST (137 mM NaCl, 2.7 mM KCl, 10 mM Na_2_HPO_4_, 2 nM KH_2_PO_4_ and 1 mM 20% Tween-20) for 2 h at room temperature (RT). An anti-His-tag mouse monoclonal antibody (1/1000 dilution, Proteintech) was used to detect the His–EsFH domain protein expressed from *E. coli* BL21 bacteria containing pET-32a-EsFH plasmid with the empty vector pET-32a as a negative control. The other membranes were incubated with anti-FOXL2 (1/500 dilution, Abcam), anti-FTZ-F1 (1/500 dilution, Santa Cruz Biotechnology), anti-DDX20 (1/1000, Proteintech) and anti-VTG (1/500, Abcam) antisera [33]. As a loading control, membranes were also incubated with anti-GAPDH monoclonal antibodies (1/1000 dilution, Proteintech). As a negative control for the specificity, the anti-FOXL2 antiserum, anti-FTZ-F1 antiserum and anti-DDX20 antiserum were further pre-adsorbed with excessive corresponding immunogen of FOXL2 (Abcam), FTZ-F1 (Proteintech) and DDX20 (Proteintech) respectively. In these negative controls, no immunostaining was observed. The membranes were washed three times with PBST (phosphate buffer solution with tween-20) for 5 min after incubation with primary antibodies at 4°C overnight. Subsequently, membranes were incubated with horseradish peroxidase (HRP)-conjugated goat anti-rabbit/mouse IgG for 1 h at RT. After three 5-min final washes with PBST, the membranes were exposed to a chemiluminescence substrate (ECL, CWBIO) according to the manufacturer's instructions.

### Immunofluorescence

Ovary tissue sections were immersion-fixed overnight in 4% paraformaldehyde in PBS and then incubated overnight in 0.5 M sucrose in PBS, placed in a cryoprotectant, cut into 10-μm thick sections and transferred to slides. After being dried at RT, the sections were boiled at 95°C–100°C for 10 min in 10 mM sodium citrate buffer (pH 6.0) for antigen retrieval and blocked in goat serum (SUNBIO) for 15 min at RT. The blocked sections were then incubated in the primary antiserum overnight at 4°C (anti-DDX20, 1:200; anti-FOXL2, 1:100; anti-FTZ-F1, 1:100; anti-VTG, 1:100). After rinsing three times with PBS for 10 min each time, the sections were exposed to FITC-conjugated secondary antibodies for FOXL2 and DDX20 or Cy3-conjugated secondary antibodies for FTZ-F1 and VTG. After washing in PBS for 10 min three times, the sections were counterstained with HelixGen Anti-fade Fluorescence Mounting Medium containing DAPI (SUNBIO), a nuclear counterstain. As a negative control, only secondary antibody was added as for the experimental sections. The sections were observed immediately under a fluorescence microscope (Leica).

### Co-immunoprecipitation

In order to corroborate the interaction between FOXL2 and DDX20 or FTZ-F1 in *E. sinensis*, co-immunoprecipitation assays were performed according to the previous method [34]. The FOXL2, DDX20, FTZ-F1 and histidine primary antibodies were added to total ovarian proteins or a mixture of total ovarian proteins and the lysate of BL21 transformed with the recombinant pET-32a-EsFH plasmid, each diluted to 20 μg/μl with lysis buffer containing PMSF and shaken slowly at 4°C overnight. Protein A/G plus-agarose beads (100 μl Santa Cruz Biotechnology) were added to capture the antigen–antibody complexes. After centrifuging at 246 ***g*** for 15 s, the supernatant was discarded and the beads were washed with PBS three times. The mixture was resuspended with 1× SDS/PAGE sample loading buffer (Beyotime) and boiled at 100°C for 5 min. The sample was centrifuged again and analysed by western blot.

### Statistical analysis

All data were calculated as means ± S.D. and evaluated by one-way ANOVA. Significance in the mean values was set at *P*<0.05.

## RESULTS

### Sequence analyses of *foxl2*, *ftz-f1* and *ddx20* cDNAs in *E. sinensis*

The full-length cDNA sequence of *foxl2* gene was determined by overlapping the 5′-RACE and 3′-RACE cDNA fragments. The complete *foxl2* cDNA was composed of a 6-nt 5′-UTR, 281-nt 3′-UTR, 1050-nt ORF (encoding a protein with 349 amino acids, predicted size of 37.846 kDa and isoelectric point of 9.72) and the termination codon TAA (Supplementary Figure S1). Multiple sequence alignment showed that the forkhead domain (DNA-binding domain), responsible for the nuclear import of this protein, was conserved among diverse species, whereas the alanine-rich region present in mammals was lacking in non-mammals (Supplementary Figure S2). In addition, FOXL2 also contains a putative nuclear localization signal (NLS, typically TRRRRMRR) at C-terminus as in vertebrate (NLS, RRRRRMKR), but the sequence is different. The phylogenetic tree indicated that the fish, amphibians, reptiles, elasmobranchs, molluscs (*Crassostrea gigas*), echinoderms (*Strongylocentrotus purpuratus*) and arthropods (*E. sinensis*) were clustered into a diverse group, but *Misgurnus anguilicaudatus* belonging to cypriniforms surprisingly was clustered with elasmobranchs (Supplementary Figure S3).

The *ftz-f1* cDNA was isolated from the testis, which spanned 1512 bp and contained an ORF encoding a protein of 504 amino acids with a predicted size of 57.4 kDa and isoelectric point of 9.72. The FTZ-F1 homologue shared typical structures of the NR5A nuclear receptor sub-family, namely the conserved DNA-binding domain containing two zinc fingers (amino acid residues 33–53 for ZF1 and 69–88 for ZF2), ligand-binding domain, F1 box and the activation function-2 (AF-2) domain (Supplementary Figure S4). Phylogenetic analysis showed that *E. sinensis* FTZ-F1 was categorized to the NR5A3 sub-family and clustered with MeFTZ-F1 (FTZ-F1 in *Metapenaeus ensis*; Supplementary Figure S5). The obtained sequence of *ddx20* spanned 1440 bp, encoding 479 amino acids (Supplemental Figure S6). The *foxl2*, *ftz-f1* and *ddx20* sequences were deposited into GenBank with accession numbers KF806733, KM657205, KF806732 respectively.

### Expression patterns of *foxl2*, *ftz-f1*, *ddx20* and *vtg* mRNAs in *E. sinensis*

**Figure 1:**
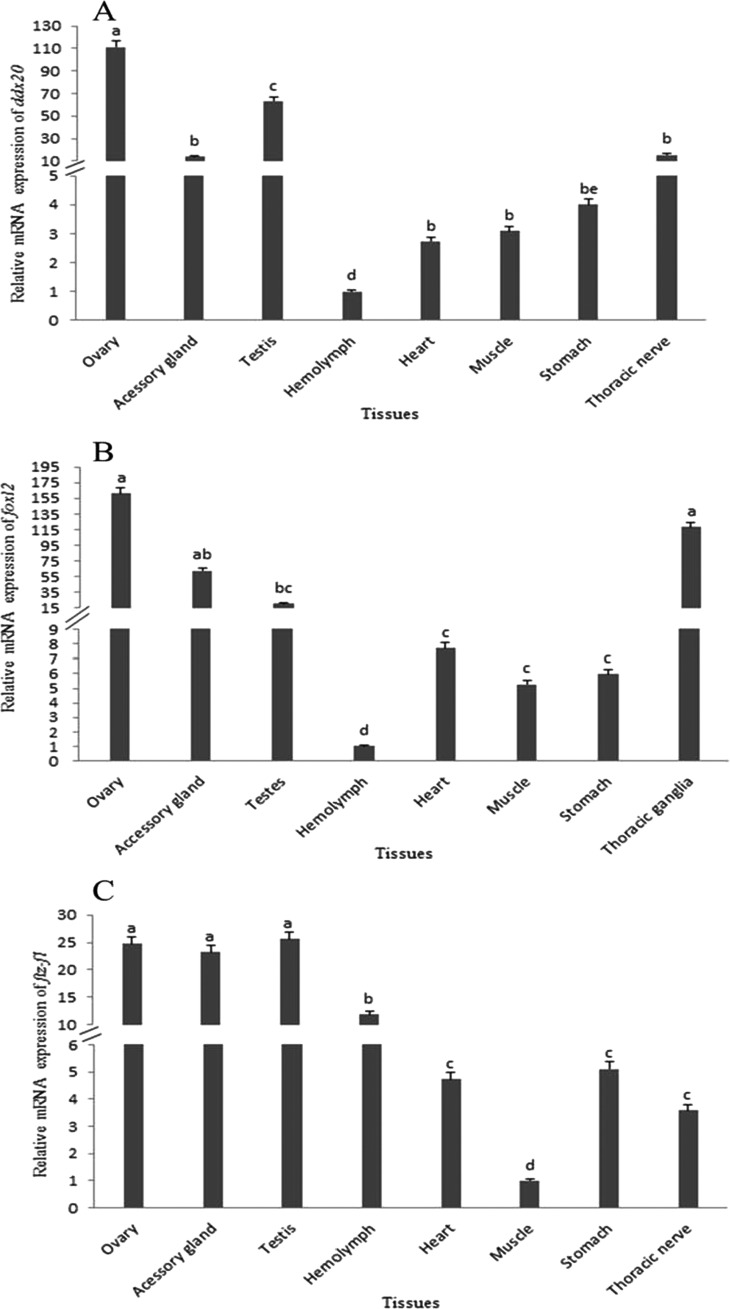
Distribution of mRNAs of *ddx20*, *foxl2* and *ftz-f1* in different tissues of *E. sinensis* detected by qRT-PCR (**A**) Relative *ddx20* mRNA expression levels in ovary, accessory gland, testis, heart, muscle, stomach and thoracic nerve was normalized to that of haemolymph. (**B**) Relative *foxl2* mRNA expression levels in ovary, accessory gland, testis, heart, muscle, stomach and thoracic nerve was normalized to that of haemolymph. (**C**) Relative *ftz-f1* mRNA expression levels in ovary, accessory gland, testis, heart, haemolymph, stomach and thoracic nerve was normalized to that of muscle.

Although qRT-PCR analysis showed that *foxl2*, *ftz-f1* and *ddx20* mRNAs were expressed in most tissues, a differential expression pattern was observed (Figure 1). The expression of *ddx20* mRNA was relatively high in the ovary and testis, relatively low in the accessory gland and thoracic nerve and very low in the haemolymph, heart, muscle and stomach (Figure 1A). The expression of *foxl2* mRNA was relatively high in ovary and thoracic nerve, relatively low in the accessory gland and testis and very low in the haemolymph, heart, muscle and stomach (Figure 1B). However, *ftz-f1* mRNA was expressed in all the tested tissues, with relatively high levels in the accessory gland, testis and ovary, relatively low levels in the haemolymph, very low levels in the heart, stomach and thoracic nerve and the lowest detectable level in muscle (Figure 1C). As expected, expression levels of *foxl2*, *ftz-f1* and *ddx20* mRNAs were relatively high in the ovary, which provided the opportunity for further study of their roles in vitellogenesis.

**Figure 2:**
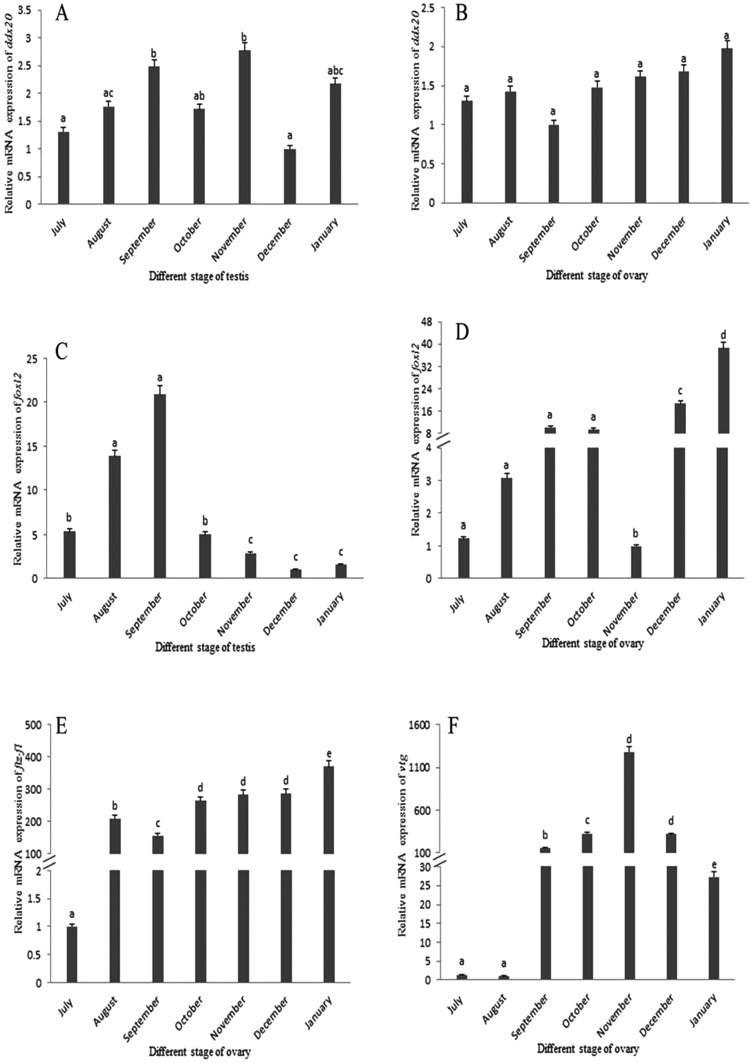
Changes in *ddx20*, *foxl2*, *ftz-f1* and *vtg* relative mRNA expression levels during germ cell development of *E. sinensis* Relative *ddx20* mRNA expression in July, August, October, November, December and January was normalized to that of December during testis development (**A**) and was normalized to that of September during ovary development (**B**). Relative *foxl2* mRNA expression was normalized to that of December during testis development (**C**) and was normalized to that of November during ovary development (**D**). Relative *ftz-f1* mRNA expression was normalized to that of July during ovary development (**E**). Relative *vtg* mRNA expression was normalized to that of August during ovary development (**F**).

During ovary and testis development, *ddx20* transcript level relatively was very high in every stage of both the testis and the ovary (Figures 2A and 2B). In the testis, relative *foxl2* transcript level was increased from July to September, decreased from October to November and decreasing continually from December to the next January (Figure 2C). However, in the ovary, relative *foxl2* mRNA levels were increased from July to September and then decreased to the lowest in November. Surprisingly, relative *foxl2* mRNA levels surged from December to the next January (Figure 2D). Relative *ftz-f1* transcripts increased significantly from July to August and then rose gradually after September until it was highly enriched in January in the ovary (Figure 2E). In ovarian tissues, the *vtg* mRNA levels relatively rose gradually from July to November, peaked in November and then dropped significantly from November to the next January (Figure 2F).

### Expression of FOXL2, FTZ-F1, DDX20 and VTG proteins in *E. sinensis*

**Figure 3:**
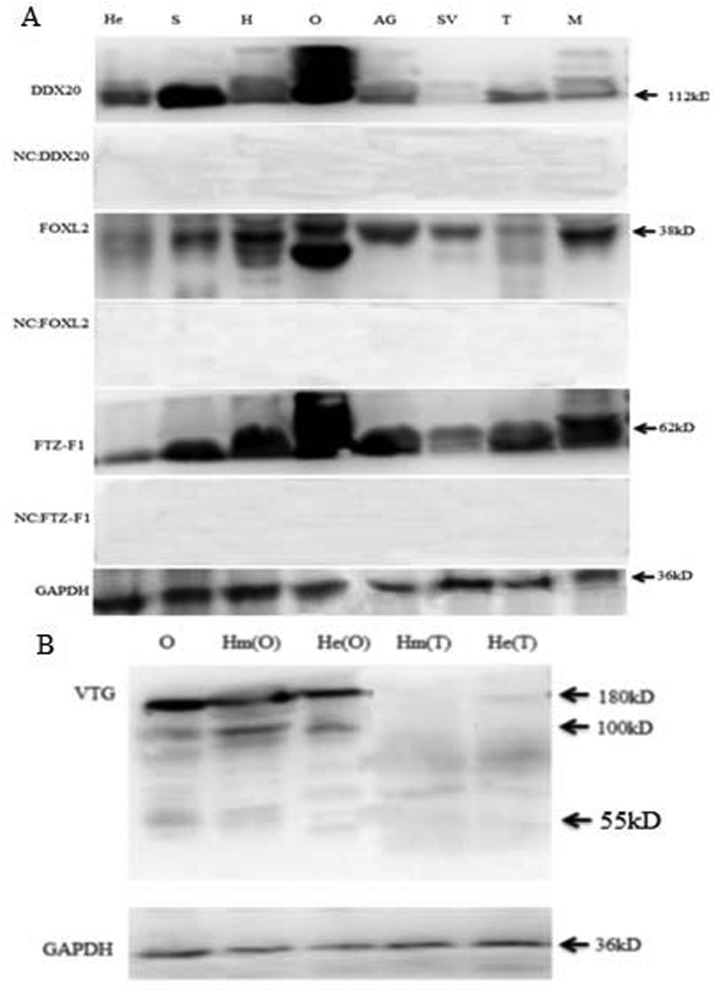
Western blot analysis of various tissues (**A**) DDX20, FOXl2 and FTZ-F1 were detected in the following tissues: AG, accessory gland; H, heart; He, hepatopancreas of male crab; M, muscle; O, ovary; S, stomach; SV, seminal vesicle; and T, testis. As a negative control, anti-FOXL2, anti-FTZ-F1 and anti-DDX20 antiserums pre-absorbed by excessive corresponding immunogens respectively. (B) VTG was detected in the following tissues: He (O), hepatopancreas of the female crab; He (T), hepatopancreas of the male crab; Hm (O), haemolymph of the female crab; Hm (T), haemolymph of the male crab; NC, negative control; O, ovary. GAPDH was detected as the internal control.

The DDX20 protein was expressed at higher levels in the ovary and stomach and at lower levels in the muscle, testis, accessory gland, heart, hepatopancreas and gill, but not in the seminal vesicle. FOXL2 and FTZ-F1 proteins were expressed in all observed tissues. FOXL2 protein was highly expressed in the muscle, ovary and heart and at a lower level in the seminal vesicle, accessory gland, stomach and gill, but hardly detectable in testis and hepatopancreas. FTZ-F1 protein was expressed at a higher level in the muscle, accessory gland, ovary, heart, stomach and gill, a lower level in testis and the lowest level in the seminal vesicle and hepatopancreas (Figure 3A). In the ovary and haemolymph of female crabs, three bands with molecular mass of ∼180, ∼100 and ∼55 kDa were detected by the anti-VTG antiserum (Figure 3B, lanes 1 and 2), whereas in the hepatopancreas of female crabs, two bands were detected with molecular masses of ∼180 and ∼100 kDa (Figure 3B, lanes 3). In the haemolymph and hepatopancreas of male crabs, no band was detected (Figure 3B, lanes 4 and 5), in line with the results that the VTG undergoes several proteolytic cleavages and generates the sub-units [35].

### Location of FOXL2, DDX20, FTZ-F1 and VTG proteins during oocyte development

**Figure 4:**
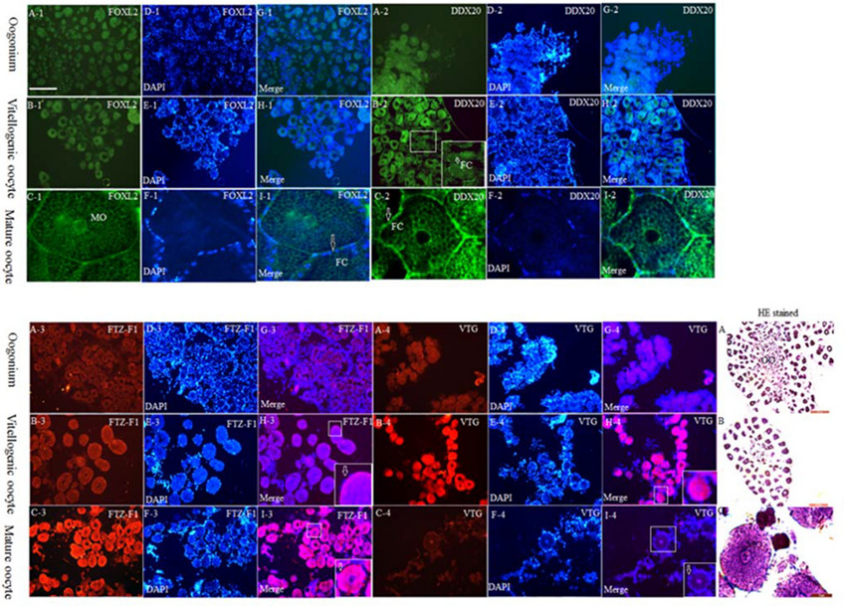
Immunofluorescence analysis of FOXL2, DDX20, FTZ-F1 and VTG proteins during ovarian development of *E. sinensis* (**A-1**–**I-1**) FOXL2; (**A-2**–**I-2**) DDX20; (**A-3**–**I-3**) FTZ-F1; (**A-4**–**I-4**) VTG. The insets in (**B-2)**, (**H-3)**, (**I-3)**, (**H-4)** and (**I-4)** are images at higher magnification of the boxed areas within each corresponding image. (**G**) Merged image of (**A**) and (**D**). (**H**) Merged image of (**B**) and (**E**). (**I**) Merged image of (**C**) and (**F**). The merged images were analysed by Image J2x software. DAPI was used to stain the nuclei blue. Haematoxylin–Eosin staining (**A**–**C**) was performed from the ovarian tissue at each stage. Abbreviations: FC, follicular cells; MO, mature oocyte; OO, oogonium. Bar=0.2 mm.

Light FOXL2 immunostaining was observed in the nucleus of the oogonium (Figure 4A-1) and vitellogenic oocyte (Figure 4B-1). At the mature stage, FOXL2 signals surged in the nucleus of follicular cells surrounding the oocyte (Figure 4C-1). At the oogonium stage, the DDX20 level was low in the nucleus of the oogonium (Figure 4A-2). At the vitellogenic oocyte stage, DDX20 signals were observed higher in the nucleus of oocyte and follicular cells (Figures 4B-2 and 4H-2). While, the DDX20 signal was only highly detected in the nucleus of follicular cells at the mature oocyte stage (Figures 4C-2 and 4I-2).

At the oogonium stage, light FTZ-F1 was detected in the nucleus of oogonium (Figure 4A-3). At the vitellogenic oocyte stage, FTZ-F1 mainly was observed in the nucleus of follicular cells and little in the nucleus of oocyte (Figure 4B-3). With the mature oocyte, FTZ-F1 was highly observed both in the nucleus of oocytes and in the follicular cells (Figure 4C-3). However, the VTG signal was low in the nucleus of the oogonium (Figure 4A-4), high in the nucleus of oocyte at the vitellogenic oocyte stage (Figure 4B-4), but barely detected in the oocyte at the mature oocyte stage (Figure 4C-4).

### FOXL2 down-regulates development of follicular cells with DDX20 and FTZ-F1 at the mature stage in *E. sinensis*

**Figure 5:**
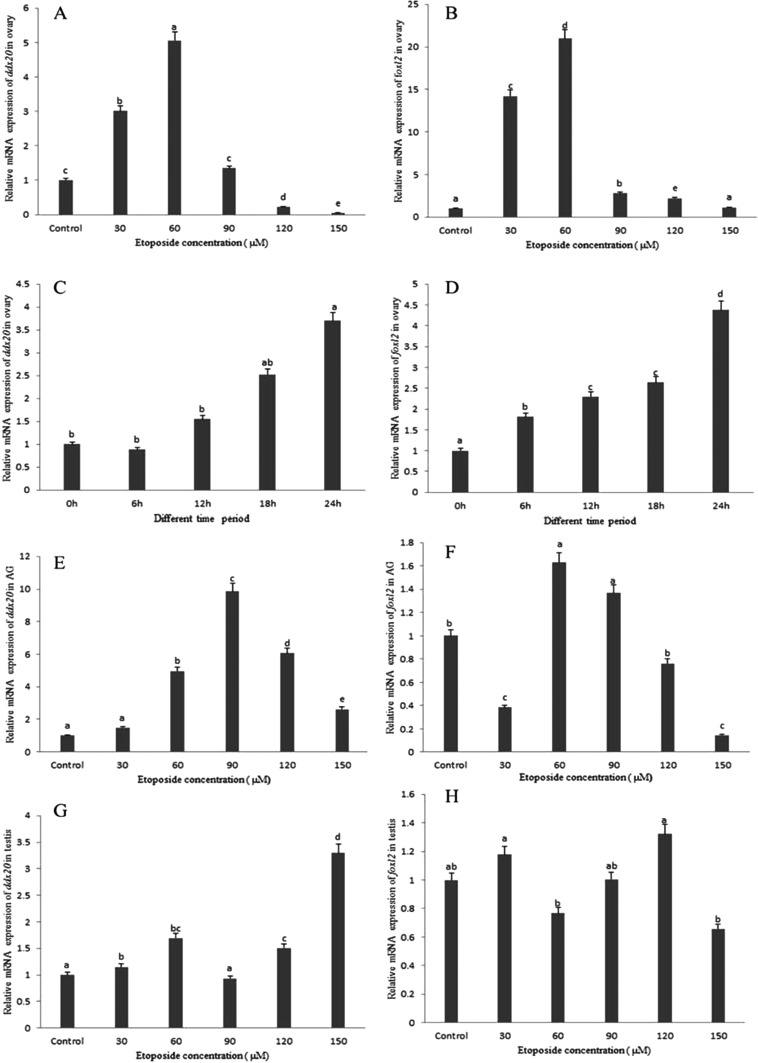
Changes in *ddx20* and *foxl2* at mRNA levels with etoposide-induced Relative *ddx20* (**A**) and *foxl2* (**B**) mRNA expression induced by different concentration of etoposide in the ovary. Relative *ddx20* (**C**) and *foxl2* (**D**) mRNA expression induced by 60 μM etoposide at different time period in the ovary. Relative *ddx20* (**E**) and *foxl2* (**F**) mRNA expression induced by different concentration of etoposide in the accessory gland. Relative *ddx20* (**G**) and *foxl2* (**H**) mRNA expression induced by different concentration of etoposide in the testis. All mRNA level was normalized to that of control.

**Figure 6:**
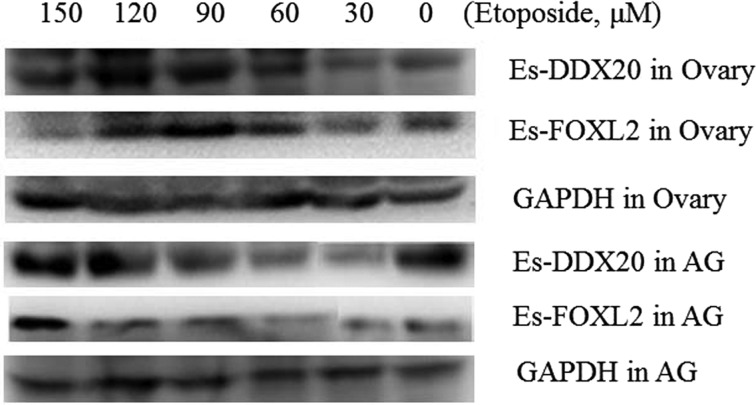
Western blot analysis of DDX20 and FOXL2 proteins expression changes in the ovary and accessory gland induced by etoposide GAPDH was detected as the internal control. Abbreviation: AG, accessory gland.

Administration of etoposide led to a significant increase, as evaluated by qRT-PCR (Figure 5) and western blot (Figure 6), in the level of DDX20 and FOXL2 in the ovary (Figures 5A–5D and 6) and in the accessory gland (Figures 5E, 5F and 6), but not in the testis (Figures 5G and 5H). Furthermore, in the ovary, expression of *ddx20* and *foxl2* mRNA increased gradually from 0 to 60 μM, peaked at 60 μM and then decreased from 60 to 150 μM (Figures 5A and 5B). As with the trend in mRNA expression, FOXL2 and DDX20 proteins also increased with increasing concentrations of etoposide, with FOXL2 reaching the peak level at 90 μM and Es-DDX20 at 120 μM (Figure 6). However, in the accessory gland, *ddx20* and *foxl2* mRNA first decreased and then increased above 30 μM of etoposide, suggesting that the mechanism between ovary and accessory gland apoptosis process was different, with the former reaching the peak at 60 μM and the latter at 90 μM (Figures 5E and 5F). At the protein level, FOXL2 increased gradually with the highest expression at 150 μM of etoposide; meanwhile, DDX20 first decreased and then increased after 30 μM, reaching the peak at 150 μM of etoposide (Figure 6).

**Figure 7:**
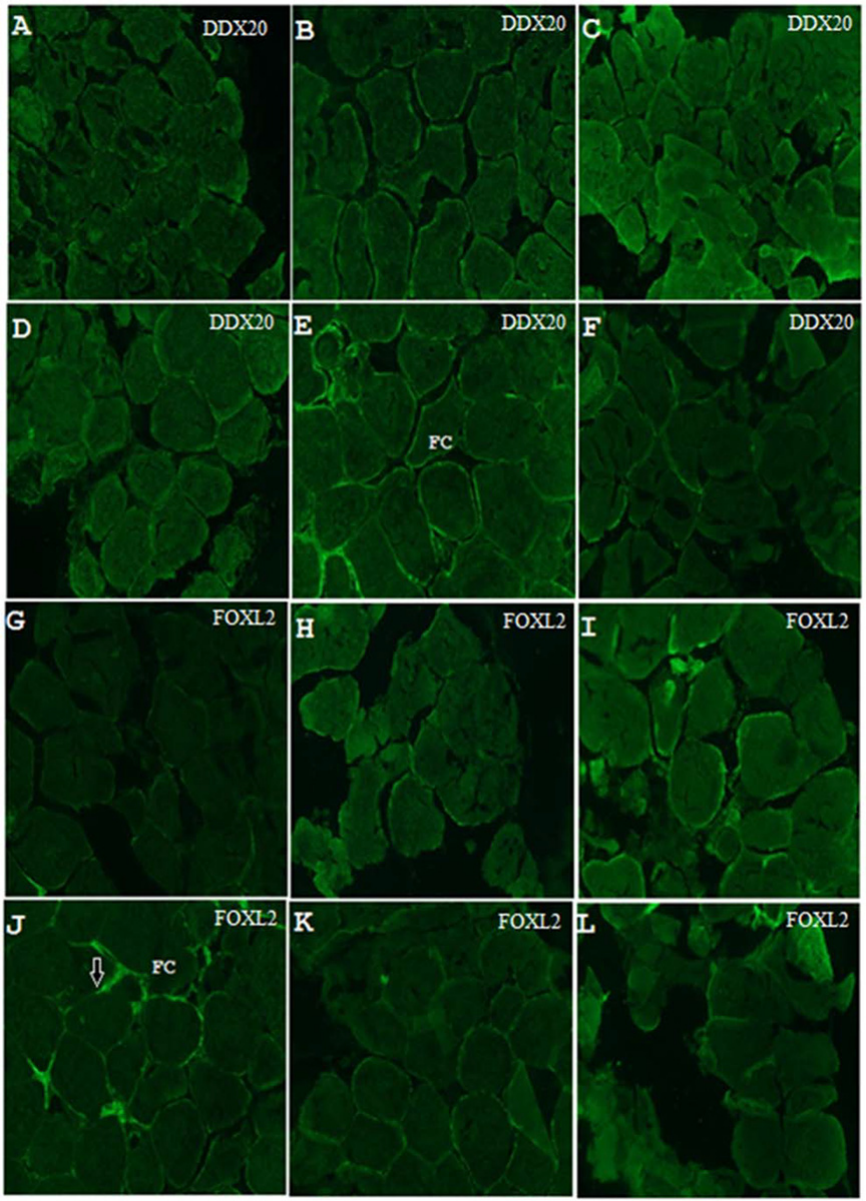
Immunofluorescence analysis of FOXL2 and DDX20 proteins expression changes in ovarian tissue culture *in vitro* induced by etoposide DDX20 protein was detected when induced by etoposide at 0 (**A**), 30 (**B**), 60 (**C**), 90 (**D**), 120 (**E**) and 150 μM (**F**). FOXL2 protein was detected when induced by etoposide at 0 (**G**), 30 (**H**), 60 (**I**), 90 (**J**), 120 (**K**) and 150 μM (**L**). Abbreviation: FC, follicular cells.

Furthermore, after primary culture of ovarian tissue *in vitro* treated with different concentration of etoposide for 24 h, the immunofluorescence result showed that levels of DDX20 and FOXL2 proteins localized in the follicular cells (Figure 7) with DDX20 reaching the peak at 120 μM (Figure 7E) and FOXL2 at 90 μM (Figure 7J), in line with the western blot analysis of the *in vivo* experiment above.

**Figure 8:**
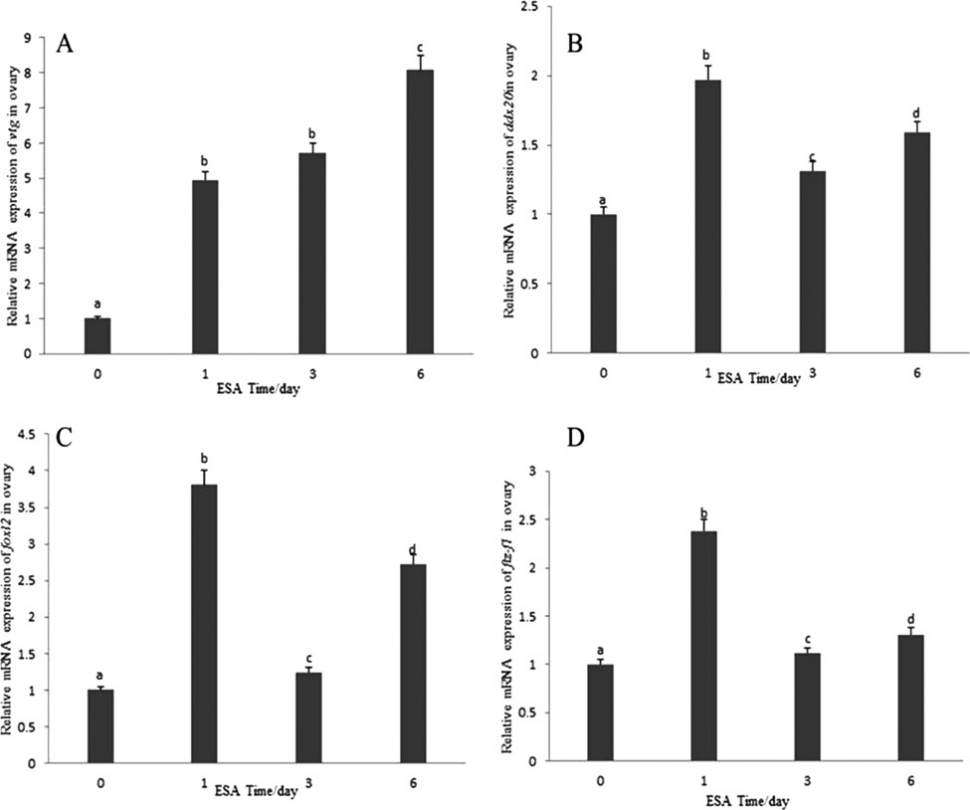
Changes in *vtg* (A), *ddx20* (B), *foxl2* (C) and *ftz-f1* (D) relative mRNA expression levels in the ovary induced by right ESA All mRNA level was normalized to that of day 0.

On the other hand, qRT-PCR of total RNA isolated from ovarian tissue of crabs subjected to ESA showed that *vtg* mRNA levels increased gradually (Figure 8A), whereas *ddx20* (Figure 8B), *foxl2* (Figure 8C) and *ftz-f1* (Figure 8D) mRNA levels significantly increased at day 1, decreased at day 3 but was still higher than the level at day 0 and increased after day 3. Interestingly, trends of *foxl2*, *ddx20* and *ftz-f1* mRNAs were synchronous.

### Identification of interaction between FOXL2 and DDX20 and the forkhead domain involved in interaction between FOXL2 and FTZ-F1 in *E. sinensis*

Gel electrophoresis was used to confirm the successful construction of the recombinant pET-32a-EsFH plasmid, which migrated at higher than 5000 bp; meanwhile, the digested recombinant plasmid presented two bands, with the larger one as the empty plasmid (>5000 bp) and the smaller one as the forkhead domain sequence (Supplementary Figure 7A). Staining of the gel with Coomassie Blue showed that the forkhead domain protein was highly induced at with 1.0 mM IPTG at 37°C for 4 h (Supplementary Figure 7B). Western blotting analysis also confirmed the presence of the forkhead domain protein in the protein lysate of BL21 transformed with recombinant pET-32a-EsFH plasmid compared with the empty plasmid (Supplementary Figure 7C).

**Figure 9:**
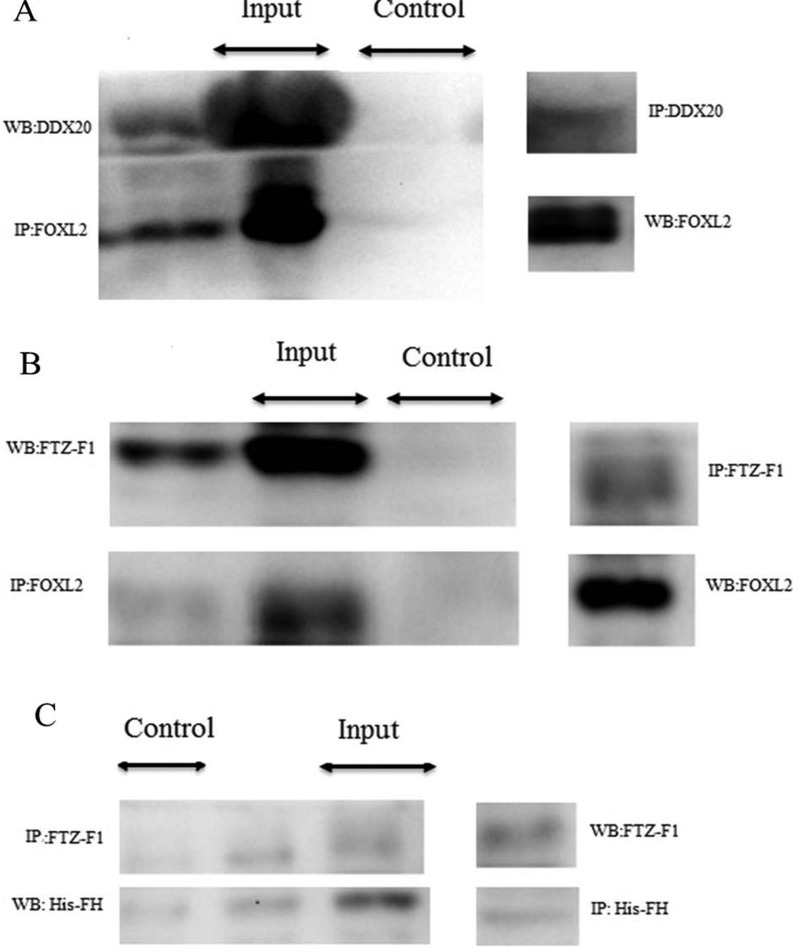
Co-Immunoprecipitation of DDX20 and FTZ-F1 with FOXL2 (**A**) FOXL2 was co-immunoprecipitated with DDX20 antibody (lane 1), vice versa (lane 4). Input: total ovary protein (**B**) FOXL2 also was co-immunoprecipitated with FTZ-F1 antibody (lane 1), vice versa (lane 4). Input: total ovary protein (**C**) FTZ-F1 was co-immunoprecipitated with histidine antibody (lane 2), vice versa (lane 4). Input: mixture of total ovary protein and BL21 of transferred with recombinant pET-32a-EsFH plasmid. Control antibody precipitation did not show any specific band. Abbreviations: IP, immunoprecipitation; WB, western blot.

Western blotting analysis of immunoprecipitated proteins with the anti-DDX20 antibody to capture the antigen–antibody complexes showed a 38-kDa band recognized by the anti-FOXL2 antibody (Figure 9A). Conversely, western blotting analysis of immunoprecipitated proteins with the anti-FOXL2 antibody showed a protein band of 112 kDa that was recognized by the anti-DDX20 antibody (Figure 9A). Similarly, the immunoprecipitate of the FOXL2 antibody contained the FTZ-F1 protein and vice versa (Figure 9B). In addition, the presence of the FTZ-F1 protein was detected in the immunoprecipitate of the histidine antibody co-incubated with the mixture of ovarian total protein and lysate of BL21 transformed with recombinant pET-32a-EsFH plasmid and vice versa (Figure 9C). To control for the specificity of the antibody used for immunoprecipitation, a non-related antibody was used to immunoprecipitate proteins instead of anti-DDX20, anti-FOXL2, anti-FTZ-F1 and anti-His antibodies, with expected no bands were detected.

## DISCUSSION

Currently, only a few recent studies have described the synthesis site of VTG in crabs. In *E. sinensis*, *vtg* mRNA observed in the female hepatopancreas and VT protein found in ovaries and hepatopancreas of vitellogenic females [5]. In the female blue crab, *Callinects sapidus*, *vtg* transcripts were found to be present only in the hepatopancreas of vitellogenic females and were not detected in the ovary at any developmental stage [35]. However, in the marine crab, *Portunus trituberculatus*, *vtg* was shown to be mainly expressed in the hepatopancreas in vitellogenesis females by Northern blotting analysis and *vtg* transcripts were also identified in the ovary [36]. In this study, VTG was observed in the hepatopancreas and ovary in *E. sinensis* (Figure 3B), which was consistent with the previous study in the land crab, *Potamon potamios* [37]. However, the molecular mechanism of VTG synthesis remains largely unknown. Based on the convincing evidence from the studies on rat and drosophila with FOXL2 [13,38], FOXL2 involved in the regulation of follicular cell apoptosis and steroid hormone pathway, which influence the VTG synthesis directly or indirectly. This made the need for fathoming the possible role of FOXL2 in regulation of VTG synthesis.

**Figure 10:**
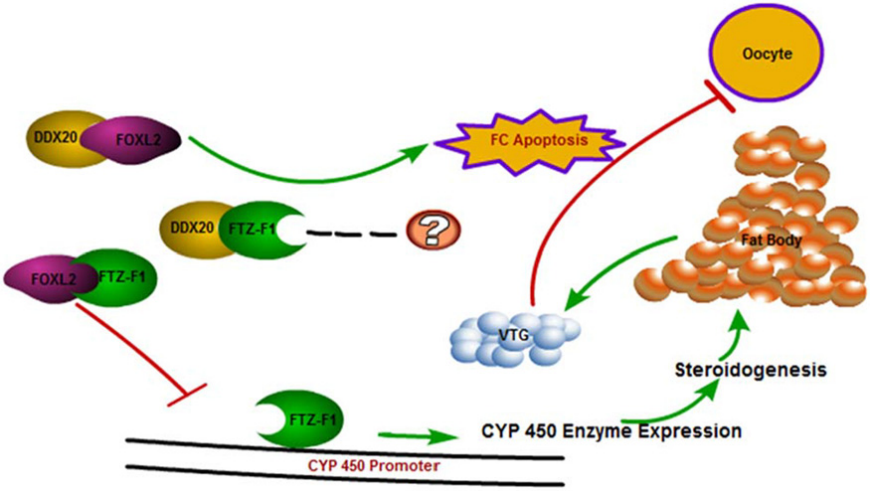
Schematic representation of VTG expression down-regulated by FOXL2 at mature stage

Our present study showed that VTG expression might be negatively correlated with FOXL2 at the mature stage (Figures 2D, 2F, 4C-1 and 4C-4), as VTG and FOXL2 have a reverse expression pattern. A finding has been reported in rat, i.e. FOXL2 could induce granulosa cells apoptosis through interacting with DDX20 by two-hybrid and co-immunoprecipitation analysis [13], which also tested and confirmed in *E. sinensis* (Figure 9A). *ddx20* and *foxl2* mRNA levels were high commonly at the mature stage of ovary (Figures 2B and 2D) when follicular cell thinned gradually and entered the apoptotic stage [6], strongly suggested that both genes involved in the regulation of follicular cell apoptosis. The spatial and temporal co-localization of these two proteins in the nucleus of follicular cell (Figures 4C-1 and 4C-2) at the mature stage further confirmed this idea. Furthermore, DDX20 and FOXL2 significantly increased with etoposide-induced apoptosis at both the mRNA (Figures 5A and 5B) and the protein levels (Figure 6) in ovary. These results were in agreement with histological observations of the ovary, in which these two proteins were primarily increased in the follicular cells (Figure 7). These results directly suggest that FOXL2 binding with DDX20 involved in the regulation of follicular cell apoptosis at the mature stage, resulted in VTG transportation to oocyte by follicular cell from haemolymph was blocked.

FOXL2 and FTZ-F1 commonly high when VTG swooped at both the mRNA and the protein levels (Figures 2D–2F, 4C-1, 4C-3 and 4C-4), which provided a clue for these two genes may also be involved in the regulation of VTG expression at the mature stage of ovary. The co-localization of FOXL2 and FTZ-F1 proteins in the nucleus of follicular cells (Figures 4C-1 and 4C-3) further provided the possibility for this hypothesis. Further, the interaction between FOXL2 and FTZ-F1 (Figure 9B) was also demonstrated as in mice [17]. The forkhead domain of FOXL2 protein is quite conserved through the vertebrates and invertebrates (Supplementary Figure S2). Hence, the present investigation further checked whether FOXL2 acts a similar fashion in crab to that in mice [17]. It was found that prokaryotic recombinant *E. sinensis* FH domain protein could bind the FTZ-F1 protein (Figure 9C).

In line with tissue patterns of FOXL2 expression in vertebrates [39], *foxl2* mRNA was highly expressed in the thoracic nerve and the ovary (Figure 1B). Furthermore, western blot analysis showed that FOXL2 protein levels were also the highest in the ovary and muscle (Figure 3A), suggesting that the main tissue targets of FOXL2 lie in the brain-pituitary-gonads (B-P-G) axis [40]. As already mentioned, the orphan nuclear receptor, Ad4BP/SF-1 (Ad4-binding protein/steroidogenic factor 1), has been found to be an important regulator of steroidogenic P450s [41–43], which stimulated fat bodies to produce VTG [16]. Since ESA removes the source of the suppressive hormones and accelerates the molting cycle and vitellogenesis, this procedure is commonly used to advance ovarian maturation. The synchronization of *ddx20*, *foxl2* and *ftz-f1* mRNA expression with ESA (Figures 8B–8D) further suggests that these three genes are involved in the regulation of VTG synthesis through the effect of steroid hormones at the mature stage. Results of the ESA experiment were consistent with the observed expression patterns of *ddx20*, *foxl2* and *ftz-f1* mRNA at the mature stage. However, to our surprising, levels of *foxl2* mRNA continue increasing with the accumulation of time after ESA, which promotes the ovary mature with expected low level of *foxl2* mRNA. In the present study, we cannot explain the reason of increased levels of *foxl2* mRNA with ESA very well, which may be due to FOXL2 is central to ovarian development and maintenance, involving in several physiological pathway [44], needed further study.

Although the levels of relative *foxl2* mRNA expression in the testis were significantly lower than that in the ovary in January (Figure 1B), high levels of relative expression in the larvae but low levels in the adult were detected in the testis of *E. sinensis* (Figures 1B, 2C and 3A), suggesting the possible involvement of FOXL2 in the steroidogenesis of even the male gonad [18] and the early development of testis [45]. The interaction domain of human DDX20 with SF-1 is also indispensable for its association with FOXL2. In humans, DDX20 (Genbank: NM_0072044, 406–825 amino acids) interacts with the proximal repression domain (PRD) of SF-1 and inhibits its transcriptional activity [46]. Although the synchronization expression pattern and co-localization of FOXL2, DDX20 and FTZ-F1 at the mature stage of ovary, whether an association between DDX20 and SF-1 exists in *E. sinensis* as in mammals and how the three proteins DDX20, FTZ-F1 and FOXL2 function in relation to ovarian maturation will require further study to fully understand the physiological mechanism of reproduction.

In conclusion, the present study is the first to demonstrate that FOXL2 down-regulate VTG synthesis not only through the regulation of follicular cell apoptosis with DDX20, but also may through the regulation of steroidogenic pathway with FTZ-F1 (Figure 10). Further studies will explore the role of FTZ-F1 in regulating the expression of cytochrome P450 enzyme.

## Abbreviations

FOXL: forkhead transcription factor
FTZ-F: fushi tarazu factor
GAPDH: glyceraldehyde-3-phosphate dehydrogenase
NLS: nuclear localization signal
POF: premature ovarian failure
qRT-PCR: quantitative real-time reverse transcription-PCR
RACE: rapid amplification of cDNA ends
RT: room temperature
SF-1: steroidogenic factor 1
VT: vitelline
VTG: vitellogenin
